# Impact of renin angiotensin system inhibitor on 3-year clinical outcomes in acute myocardial infarction patients with preserved left ventricular systolic function: a prospective cohort study from Korea Acute Myocardial Infarction Registry (KAMIR)

**DOI:** 10.1186/s12872-021-02070-x

**Published:** 2021-05-21

**Authors:** Kyung-Hee Kim, Byoung Geol Choi, Seung-Woon Rha, Cheol Ung Choi, Myung-Ho Jeong, Other KAMIR-NIH investigators, Seung-Woon Rha, Tae Hoon Ahn, Junghan Yoon, Hyo-Soo Kim, Ki-Bae Seung, Hyeon-Cheol Gwon, Shung Chull Chae, Chong-Jin Kim, Kwang Soo Cha, Jung-Hee Lee, Jei Keon Chae, Seung-Jae Joo, Chang-Hwan Yoon, Seung-Ho Hur, In-Whan Seong, Kyung-Kuk Hwang, Doo-Il Kim, SeokSeok Kyu Oh, Jin-Yong Hwang, Myung Ho Jeong

**Affiliations:** 1 Cardiovascular Center, Incheon Sejong Hospital, Incheon, South Korea; 2 Cardiovascular Research Institute, University, Seoul, South Korea; 3 Cardiovascular Center, Korea University Guro Hospital, 148, Gurodong-ro, Guro-gu, Seoul, 08308 South Korea; 4 Division of Cardiology, Department of Medicine, Chonnam National University Hospital, Gwangju, South Korea

**Keywords:** RAS inhibitor, AMI, Preserved LV systolic function

## Abstract

**Background:**

Patients with acute myocardial infarction (AMI) are usually treated with angiotensin-converting enzyme inhibitors (ACEIs), or angiotensin receptor blockers (ARBs) if ACEIs are not tolerated. However, there is no data regarding the impact of switching from ACEIs to ARBs on long-term clinical outcomes in AMI patients with preserved left ventricular (LV) systolic function especially beyond 1 year. To investigate the effectiveness of treatment with ACEIs or ARBs on clinical outcomes over 3 years in AMI patients with preserved LV systolic function following percutaneous coronary intervention.

**Method:**

It is a prospective cohort study using data from a nationwide large scale registry with 53 hospitals involved in treatment of acute myocardial infarction (AMI) in Korea. Between March 2011 and September 2015, we enrolled 6236 patients with AMI who underwent primary percutaneous coronary intervention and had a left ventricular ejection fraction ≥ 50%. Main outcome measures composite of total death or recurrent AMI over 3 years after AMI. Patients were divided into an ACEI group (n = 2945), ARB group (n = 2197), or no renin-angiotensin system inhibitor (RASI) treatment (n = 1094). We analyzed patients who changed treatment. Inverse probability of treatment weighting (IPTW) analysis was also performed.

**Results:**

After the adjustment with inverse probability weighting, the primary endpoints at 1 year, AMI patients receiving ACEIs showed overall better outcomes than ARBs [ARBs hazard ratio (HR) compared with ACEIs 1.384, 95% confidence interval (CI) 1.15–1.71; *P* = 0.003]. However, 33% of patients receiving ACEIs switched to ARBs during the first year, while only about 1.5% switched from ARBs to ACEIs. When landmark analysis was performed from 1 year to the end of the study, RASI group showed a 31% adjusted reduction in primary endpoint compared to patients with no RASI group (HR, 0.74; 95% CI 0.56–0.97; *P* = 0.012).

**Conclusions:**

This result suggests that certain patients got benefit from treatment with ACEIs in the first year if tolerated, but switching to ARBs beyond the first year produced similar outcomes. RASI beyond the first year reduced death or recurrent AMI in AMI patients with preserved LV systolic function.

*CRIS Registration number*: KCT0004990.

## Key points

- *Question*: Do renin angiotensin system inhibitors have long term clinical beneficial effect in acute myocardial infarction (AMI) patients with preserved left ventricular systolic function?
- *Findings*: In this prospective cohort study including 6236 AMI patients who underwent percutaneous coronary intervention, 33% of patients receiving angiotensin converting enzyme inhibitors switched to angiotensin receptor blockers during the first year. Renin angiotensin system inhibitors (RASI) beyond the first year reduced death or recurrent AMI in AMI patients with preserved left ventricular systolic function.
- *Meaning*: Switching the medication of RASI does not impact on long term major clinical outcomes in AMI patients with preserved left ventricular systolic function. Continuation of treatment is necessary.

## Introduction

Angiotensin-converting enzyme inhibitors (ACEIs) are considered as primary drugs for the secondary prevention of myocardial infarction (MI), and angiotensin receptor blockers (ARBs) are used when ACEIs are not tolerated [1, 2]. Both these types of drugs are recognized as renin-angiotensin system inhibitors (RASI), but differences in their modes of action have been reported to be associated with clinically different outcomes.

The usefulness of ACEIs for secondary prevention of cardiovascular events in patients with acute myocardial infarction (AMI) and heart failure with reduced ejection fraction (HFrEF) has been established by many randomized controlled trials (RCTs) [3–5]. However, the effectiveness of ACEIs and ARBs as first-line drugs in patients with AMI with preserved left ventricular (LV) function has not been established. While both classes are equally effective in patients who have vascular disease or high-risk diabetes but do not have heart failure, they may be differentially beneficial in MI patients with preserved LV systolic function [6]. Additionally, while many physicians use ACEIs as a first-line therapy, up to 20% of patients develop adverse reactions such as a cough and/or angio-edema, and change to ARBs [7], but no data is available indicating whether changing from ACEIs to ARBs in MI patients with preserved LV systolic function can affect long-term clinical outcomes including mortality.

We sought to investigate the effectiveness of treatment with ACEIs or ARBs on clinical outcomes over 3 years in AMI patients with preserved LV systolic function following percutaneous coronary intervention (PCI) from a nationwide large-scale registry of AMI patients in South Korea. We also investigated how many patients changed medication after 1 year and the impact of changing medication on long-term outcomes.

## Methods

### Study population

The study population was enrolled from the Korea AMI registry (KAMIR)-National Institutes of Health (NIH) registry. The design of KAMIR study has been described in our previous studies [8, 9] and the details of the registry can be found at the KAMIR website (http://www.kamir.or.kr). Briefly, it is a prospective, multicenter online registry designed to reflect the “real world” practice in a series of Korean AMI patients since November 2005 to investigate the current clinical outcomes. Participating centers have a high volume of patients and have facilities for primary PCI and onsite cardiac surgery. From November 2011 to December 2015, a total of 13,104 patients with AMI have been enrolled in a nationwide KAMIR-NIH registry. A trained study coordinator, independent research personnel collected clinical, laboratory, and clinical outcome data using a standardized case report form and protocol. Each patient was followed up during an outpatient clinic visit or contacted by telephone conversation. Inclusion criteria for the present analysis were consecutive patients aged ≥ 18; ST segment elevation or non-ST segment elevation myocardial infarction; and patients undergoing PCI. The investigators defined AMI as the criteria for the universal definition of myocardial infarction [10]. We excluded patients who died in hospital; lacked documentation of drugs prescribed at discharge and 1-year after discharge; concomitantly used an ACEI and ARB; or had a LV ejection fraction (EF) < 50% or lacked information on LVEF. From registered patients, we ultimately included 6236 in this analysis. Patients were divided into the ACEIs group and the ARBs group or the no RASI group according to the use of ACEI or ARB at discharge.

### Percutaneous coronary intervention procedure and medical treatment

Physicians performed coronary angiography and stent implantation according to standard techniques and the type of the stent chosen was at the operator's discretion [11]. All patients received a 300 mg loading doses of aspirin and a 300–600 mg loading of clopidogrel or 60 mg of prasugrel or 90 mg of ticagrelor before the procedure, unless they had previously received these antiplatelet drugs. Periprocedural anticoagulation was administered according to standard regimens. Most of the patients maintained dual antiplatelet therapy at the time of PCI or discharge, and the duration of dual antiplatelet therapy was determined by the physicians. The decision to use glycoprotein IIb/IIIa inhibitors and to perform thrombus aspiration or intravascular ultrasonography was at the operator’s discretion. Drug-eluting stents (DES) were deployed after prior balloon angioplasty. During the in-hospital period, the patients received cardiovascular beneficial medications including beta-blockers, ACEI, ARB, calcium channel blockers, and statins. After discharge, the patients were encouraged to maintain the same medication they received during the hospitalization but some patients switched ACEI to ARB or ARB to ACEI and unexpectedly, some patients did not use RASI after 1 year.

### Study definition and endpoint

A successful PCI was defined as normal antegrade coronary blood flow is achieved in > 95% of patients without major adverse cardiac events (MACE) in the presence of thrombolysis in myocardial infarction (TIMI) flow grade 3 [12, 13]. The key combined primary endpoint was as defined as the composite of all-cause death or recurrent AMI. The key combined secondary endpoint was MACE, as defined as the composite of all-cause death, recurrent AMI, and any repeat revascularization. Also, the secondary endpoints were the occurrence of any clinical events such as all-cause death, recurrent AMI, any repeat revascularization including surgical coronary artery bypass graft (CABG), or repeat PCI, stroke, and re-hospitalization due to heart failure (HF). All deaths are considered to be cardiac in origin unless a non-cardiac origin was definitely documented. Recurrent MI is defined as recurrent symptoms with new ST-segment elevation or re-elevation of cardiac markers to at least twice the upper limit of normal [13]. Target lesion revascularization (TLR) is defined as repeat PCI within the index procedure stent or 5 mm edge. Target vessel revascularization (TVR) is defined as any repeat PCI or surgical CABG of any segment in the target vessel. Any repeat revascularization is defined as any repeat PCI or CABG of the target vessel or non-target vessel. Target lesion failure is defined as the composite of clinically driven TLR, recurrent AMI, or cardiac death related to the target vessel [14]. All participants are required to visit the outpatient department of cardiology at the end of the first month and then every three to six months after the PCI procedure, as well as whenever angina-like symptoms occurred [15].

### Ethics statement

This study was conducted in compliance with the Declaration of Helsinki regarding investigations in humans, and the study protocol was reviewed and approved by the each Institutional Review Board (IRB) of participating hospitals (Chonnan National Hospital, IRB 2011-E63002-00, 2012-E63003-00, 2013-E63005-00, 2014-E63005-01, 2013-E63005-02). Written informed consent was obtained from each patient when they were enrolled. This registry has not been registered on ClinicalTrials.gov due to observational study.

### Statistical analysis

For continuous variables, differences among the three groups are evaluated using the one-way ANOVA or Kruskal–Wallis test. Data are expressed as mean ± standard deviations. For discrete variables, differences are expressed as counts and percentages and analyzed with the χ^2^ or Fisher’s exact test among the three groups. To adjust for any potential confounders, an inverse probability of treatment weighting (IPTW) analysis is performed [16]. We utilized generalized boosted models to estimate the propensity score weight of each treatment using methods developed for comparison of multiple treatments.

The average treatment effect on the population weights was estimated using the multinomial propensity scores (MNPS) function in the Twang package (http://www.rand.org/statistics/twang/downloads.html/mnps_turorial_code.r.) in R Statistical Software (R Foundation for Statistical Computing, Vienna, Austria). We tested all available variables that could be of potential relevance: age, sex (male), LVEF, cardiovascular risk factors (e.g., hypertension, diabetes, dyslipidemia, stroke), co-medication treatment (e.g., aspirin, other anti-platelets, calcium channel blockers, beta-blockers, and statins), angiographic and procedural characteristics (e.g., target vessel, number of diseased vessels, and DES type). Various clinical outcomes for up to 3 years are estimated by Cox-proportional hazards models analysis. Binary logistic regression analysis is used to assess the hazard ratio (HR) of the ACEIs group compared to the ARBs group and the non-RASI user group in the IPTW population. For all analyses, a two-sided *P* < 0.05 was considered statistically significant. All data are processed with SPSS (version 24.0, SPSS-PC, Inc. Chicago, Illinois) and R statistical software.

## Results

### Baseline and procedural characteristics

#### Overall population

The baseline, laboratory, angiographic, and procedural characteristics of the study population are summarized in Table 1. Among the 6236 eligible patients, at discharge ACEIs were prescribed to 2945 patients (47%) and ARBs to 2197 patients (35%). Renin angiotensin system blockers were not prescribed to 1094 patients (18%). Compared with patients receiving ACEIs, those in the group receiving ARBs were on average older, had a higher rate of diabetes mellitus (most of which was well controlled), had a lower rate of right coronary artery as the infarct-related artery, and had a lower rate of multi-vessel disease. Overall, patients who did not receive RASI, apart from having a slightly higher rate of low blood pressure, were on average not higher-risk patients when compared to patients in the group which received RASI. In addition, patients who did not receive RASI were less likely to have received β blockers and statins, but more likely to have received Ca-channel blockers.

**Table 1:** Baseline clinical, angiographic and procedural characteristics of acute myocardial infarction patients with preserved left ventricular systolic function according to treatment at discharge

Variables, n (%)	Entire population				Matched population			
	ACEI (n = 2945)	ARB (n = 2197)	No RASI (n = 1094)	*P* value	ACEI (n = 5854)	ARB (n = 5691)	No RASI (n = 5630)	*P* value
Sex, male	2315 (78.6%)	1637 (74.5%)	869 (79.4%)	< 0.001	77.9%	77.2%	78.3%	0.360
Age, year	61.9 ± 12.2	63.5 ± 12.1	63.2 ± 12.2	< 0.001	62.6 ± 12.2	62.9 ± 12.2	62.6 ± 12.0	0.468
LV ejection fraction, %	58.1 ± 5.8	59.4 ± 6.1	58.5 ± 6.0	< 0.001	58.5 ± 5.9	58.6 ± 5.9	58.7 ± 5.9	0.313
Blood pressure, mmHg								
Systolic BP	135 ± 27	132 ± 26	127 ± 25	< 0.001	135 ± 27	132 ± 26	128 ± 25	< 0.001
Diastolic BP	81 ± 16	80 ± 16	77 ± 15	< 0.001	81 ± 16	80 ± 16	78 ± 15	< 0.001
Heart rate, bpm	74 ± 16	76 ± 16	74 ± 17	0.009	74 ± 16	75 ± 16	74 ± 17	< 0.001
Final diagnosis								
STEMI	1435 (48.7%)	871 (39.6%)	514 (46.9%)	< 0.001	45.7%	44.7%	45.9%	0.395
Non-STEMI	1510 (51.2%)	1326 (60.3%)	580 (53.0%)	< 0.001	54.2%	55.2%	54.0%	0.395
Risks of patients								
Hypertension	1388 (47.1%)	1230 (55.9%)	482 (44.0%)	< 0.001	49.3%	50.3%	48.7%	0.249
Diabetes mellitus	660 (22.4%)	627 (28.5%)	284 (25.9%)	< 0.001	24.3%	25.9%	25.7%	0.104
Dyslipidemia	364 (12.3%)	284 (12.9%)	142 (12.9%)	0.786	12.8%	12.4%	13.3%	0.332
Kidney disease	358 (12.2)	322 (14.7)	162 (14.8)	0.013	12.4%	13.6%	13.3%	0.115
eGFR-MDRD (mL/min/1.73m^2^)	91.6 ± 39.8	91.5 ± 31.4	91.2 ± 45.9	0.959	91.6 ± 41.4	92.6 ± 30.8	91.7 ± 41.6	0.304
Stroke	118 (4.0%)	99 (4.5%)	51 (4.6%)	0.551	4.1%	4.1%	4.2%	0.937
Ischemic	13 (0.4%)	14 (0.6%)	7 (0.6%)	0.574	0.5%	0.5%	0.7%	0.258
Hemorrhagic	108 (3.6%)	88 (4.0%)	44 (4.0%)	0.780	3.7%	3.7%	3.5%	0.830
Smokers								
Current	1304 (44.2%)	845 (38.4%)	447 (40.8%)	< 0.001	41.7%	41.3%	41.6%	0.906
Ex-smokers	548 (18.6%)	487 (22.1%)	218 (19.9%)	0.007	20.3%	20.7%	19.5%	0.270
Laboratory findings								
Glucose	158 ± 67	161 ± 70	161 ± 77	0.543	158 ± 66	161 ± 71	161 ± 80	0.074
HbA1c	6.3 ± 1.3	6.4 ± 1.4	6.4 ± 1.4	0.032	6.3 ± 1.3	6.4 ± 1.4	6.4 ± 1.4	0.654
*Angiographic and procedural characteristics*								
Multi-vessel disease	1512 (51.3%)	1004 (45.6%)	551 (50.3%)	< 0.001	49.1%	48.0%	49.5%	0.270
Target vessel								
LAD	1928 (65.4)	1370 (62.3)	703 (64.2)	0.071	64.2%	63.6%	65.3%	0.191
LCX	1345 (45.6)	954 (43.4)	463 (42.3)	0.097	44.4%	43.9%	42.2%	0.051
RCA	1694 (57.5)	1128 (51.3)	644 (58.8)	< 0.001	55.4%	54.6%	55.9%	0.389
Left main	121 (4.1)	87 (3.9)	47 (4.2)	0.898	3.9%	3.4%	4.1%	0.177
Drug-eluting stents								
Everolimus-	1501 (50.9%)	1098 (49.9%)	525 (47.9%)	0.240	50.0%	50.9%	49.2%	0.220
Zotarolimus-	773 (26.2)	477 (21.7)	276 (25.2)	0.001	25.4%	23.4%	25.4%	0.981
Biolimus A9	558 (18.9%)	534 (24.3%)	228 (20.8%)	< 0.001	20.4%	21.5%	21.2%	0.280
Sirolimus-	100 (3.3%)	79 (3.5%)	59 (5.3%)	0.010	3.7%	3.6%	3.6%	0.851
Paclitaxel-	6 (0.2%)	1 (0.0%)	1 (0.0%)	0.273	0.1%	0.0%	0.1%	0.507
Number of stents	1.16 ± 0.40	1.17 ± 0.42	1.17 ± 0.43	0.389	1.16 ± 0.41	1.16 ± 0.42	1.17 ± 0.42	0.946
Stent diameter, mm	3.17 ± 0.44	3.14 ± 0.44	3.14 ± 0.45	0.020	3.16 ± 0.44	3.15 ± 0.44	3.16 ± 0.44	0.257
Total stent length, mm	29.1 ± 12.7	28.1 ± 12.9	28.6 ± 12.9	0.001	29.0 ± 13.0	28.7 ± 12.8	28.4 ± 12.2	0.381
Discharge medication								
Aspirin	2931 (99.5%)	2184 (99.4%)	1083 (98.9%)	0.156	99.5%	99.5%	99.3%	0.260
Clopidogrel	2073 (70.3%)	1404 (63.9%)	693 (63.3%)	< 0.001	67.1%	66.5%	66.3%	0.626
Prasugrel	302 (10.2%)	285 (12.9%)	118 (10.7%)	0.008	11.1%	11.5%	10.9%	0.593
Ticargrelor	554 (18.8%)	497 (22.6%)	268 (24.4%)	< 0.001	21%	21.3%	21.7%	0.623
Ca-channel blockers	136 (4.6%)	180 (8.1%)	117 (10.6%)	< 0.001	6.7%	7.0%	7.3%	0.485
Beta-blockers	2741 (93.0%)	1921 (87.4%)	757 (69.1%)	< 0.001	87.8%	86.9%	86.3%	0.053
Statin	2848 (96.7%)	2114 (96.2%)	1028 (93.9%)	< 0.001	96.5%	96.5%	95.7%	0.057

RASI, renin angiotensin system inhibitor; IPTW, inverse probability of treatment weighting; LV, left ventricular; ACEI, angiotensin converting enzyme inhibitor; ARB, angiotensin receptor blocker; STEMI, ST segment elevation myocardial infarction; LAD, left anterior descending artery; LCX, left circumflex artery, RCA = right coronary artery

After adjustment using inverse probability of treatment weighting (IPTW), there were no statistically significant differences in baseline clinical, angiographic, and procedural characteristics among the different groups (ACEIs, ARBs, and no RASI treatment), with the exception of blood pressure (Table 1). However, the observed difference in systolic blood pressure among the groups was small, and only varied by 10-15 mmHg. Most of patients had blood pressure above 100 mmHg, which is above key shock thresholds for concern.

### Major clinical outcomes

#### Overall population at 1 year and 3 years after AMI

Table 2 shows the cumulative incidences of the major clinical outcomes during the 3-year follow-up period of the study groups. Total death or recurrent AMI occurred in 175 patients (5.9%) in the ACEI group, 164 patients (7.4%) in the ARB group (*P* = 0.03 for comparison with ACEIs) and 93patients (8.5%) in the no RASI group (*P* = 0.004 for ACEIs). Although the primary composite endpoints of ARB group seemed to be numerically lower than that of no RASI group, long-term outcomes were not statistically different between crude population (*P* = 0.297). When IPTW was performed at the primary endpoint (total death or recurrent AMI) after 3 years of AMI, patients receiving ACEIs had overall better outcomes than patients receiving ARBs (ACEi 6.0% vs. ARBs 7.0%, *P* = 0.033). Between ARBs and no RASI group, the primary endpoint were not significantly different (ARB 7.0% vs no ACEI/ARB group 7.3%, *P* = 0.553). However, the incidence of total death was significantly lower in the ARBs group (ARB 4.4% vs. no RASI 5.2%, *P* = 0.048).

**Table 2:** Clinical outcomes of acute myocardial infarction patients with preserved left ventricular systolic function according to discharge treatment during a 3-year follow-up

Variables, n (%)	ACEI	ARB	No RASI	*P* value			
				All groups	ACE vs. ARB	ACE vs. No RASI	ARB vs. No RASI
Entire population	(n = 2945)	(n = 2197)	(n = 1094)				
Primary endpoint at 1 year	175 (5.9%)	164 (7.4%)	93 (8.5%)	0.008	0.030	0.004	0.297
Total death	110 (3.7%)	104 (4.7%)	67 (6.1%)	0.004	0.076	0.001	0.090
Myocardial infarction	71 (2.4%)	70 (3.1%)	30 (2.7%)	0.242	0.092	0.549	0.485
Secondary endpoints							
MACE	377 (12.8%)	289 (13.1%)	169 (15.4%)	0.083	0.709	0.029	0.073
Revascularization	268 (9.1%)	171 (7.7%)	103 (9.4%)	0.163	0.095	0.758	0.110
Target lesion: TLR	72 (2.4%)	59 (2.6%)	35 (3.1%)	0.415	0.588	0.185	0.405
Target vessel: TVR	137 (4.6%)	102 (4.6%)	64 (5.8%)	0.244	0.988	0.120	0.136
Non-TVR	139 (4.7%)	73 (3.3%)	40 (3.6%)	0.033	0.013	0.144	0.621
Stroke	44 (1.4%)	57 (2.5%)	19 (1.7%)	0.016	0.005	0.580	0.123
Hemorrhagic	11 (0.3%)	21 (0.9%)	4 (0.3%)	0.014	0.009	> 0.999	0.066
Ischemic	31 (1.0%)	34 (1.5%)	13 (1.1%)	0.281	0.116	0.712	0.413
TIA	3 (0.1%)	2 (0.0%)	2 (0.1%)	0.740	> 0.999	0.617	0.604

Variables, n (%)	ACEI	ARB	No RASI	*P* value			
				All groups	ACE vs. ARB	ACE vs. No RASI	ARB vs. No RASI
IPTW weighted population	(n = 5854)	(n = 5691)	(n = 5630)				
Primary endpoint at 3 years	6.0%	7.0%	7.3%	0.018	0.033	0.007	0.553
Total death	3.9%	4.4%	5.2%	0.005	0.213	0.001	0.048
Myocardial infarction	2.3%	3.0%	2.5%	0.036	0.012	0.416	0.095
Secondary endpoints							
MACE	12.7%	13.2%	13.8%	0.244	0.503	0.095	0.321
Revascularization	8.9%	8.2%	8.8%	0.382	0.207	0.917	0.251
Target lesion: TLR	2.5%	2.6%	2.6%	0.955	0.806	0.780	0.973
Target vessel: TVR	4.8%	4.6%	4.9%	0.750	0.620	0.797	0.455
Non-TVR	4.3%	3.7%	3.9%	0.301	0.126	0.356	0.548
Stroke	1.6%	2.5%	1.7%	0.002	0.001	0.675	0.006
Hemorrhagic	0.3%	0.9%	0.4%	< 0.001	0.000	0.323	0.003
Ischemic	1.2%	1.4%	1.0%	0.106	0.290	0.279	0.034
TIA	0.0%	0.0%	0.2%	0.028	> 0.999	0.031	0.037

RASI, renin angiotensin system inhibitor, IPTW, inverse probability of treatment weighting; LV, left ventricular; ACEI, angiotensin converting enzyme inhibitor; ARB, angiotensin receptor blocker; MACE, major adverse cardiovascular event; STEMI, ST segment elevation myocardial infarction; TIA, transient ischemic attack

When Cox regression analysis with IPTW was performed at the primary end point (total death or recurrent AMI) after 1 year of AMI, patients receiving ACEIs showed overall better outcomes than patients receiving ARBs (ARBs compared with ACEIs, hazard ratio [HR] 1.384, 95% confidence interval [CI] 1.15–1.717, *P* = 0.003, Table 3). There was no difference in the incidence of total death between patients in the two groups (ARBs compared with ACEIs, HR1.308, 95% CI 0.999–1.714, *P* = 0.051). However, when solely examining, patients receiving ACEIs were less likely to experience recurrent AMI compared to patients receiving ARBs (ARBs compared with ACEIs, HR 1.577, 95% CI 1.127–2.205, *P* = 0.008). We also observed that patients receiving ARBs were twice more likely to experience a stroke compared to the group receiving ACEIs (ARBs compared with ACEIs, HR 2.015, 95% CI 1.391–2.917, *P* < 0.001) at 1 year after AMI. Both ARB group and no RASI group showed similar risk of cardiac death or myocardial infarction (no RASI group compared with ARBs, HR 1.182, 95% CI 0.974 to 1.435; *P* = 0.0.089) after adjustment with IPTW at 1 year after AMI. However, treatment with ARBs was associated with a significant lower incidence of cardiac death compared with no RASI (no RASI group compared with ARBs, HR 1.182, 95% CI 0.974 to 1.435; *P* = 0.002). These results were similar in the 3-year follow up after AMI. Receiving ACEIs had overall better outcomes than patients receiving ARBs or no RASI. Receiving ARBs have numerically lower incidence of primary endpoint compared with no RASI group but statistically significantly lower incidence of total death at 3 years after AMI.

**Table 3:** Comparative unadjusted Cox-regression hazard ratios of death or myocardial infarction in Entire and IPTW populations according to discharge treatment

	Unadjusted		Matched Population	
	HR (95% CI)	*P* value	HR (95% CI)	*P* value
*One year clinical outcomes*				
Primary endpoint (Death or MI)				
ACEI vs. ARB	1.449 (1.045–2.008)	0.026	1.384 (1.115–1.717)	0.003
ACEI vs. No RASI	1.991 (1.384–2.864)	< 0.001	1.637 (1.328–2.019)	< 0.001
ARB vs. No RASI	1.373 (0.959–1.968)	0.083	1.182 (0.974–1.435)	0.089
All-cause death				
ACEI vs. ARB	1.408 (0.932–2.128)	0.103	1.308 (0.999–1.714)	0.051
ACEI vs. No RASI	2.509 (1.633–3.853)	< 0.001	1.895 (1.473—2.439)	< 0.001
ARB vs. No RASI	1.781 (1.164–2.725)	0.008	1.448 (1.145–1.831)	0.002
Myocardial infarction: MI				
ACEI vs. ARB	1.573 (0.962–2.569)	0.070	1.577 (1.127–2.205)	0.008
ACEI vs. No RASI	1.259 (0.665–2.384)	0.479	1.351 (0.955–1.911)	0.089
ARB vs. No RASI	0.800 (0.428–1.494)	0.485	0.856 (0.627–1.169)	0.331
Stroke				
ACEI vs. ARB	2.254 (1.308–3.882)	0.003	2.015 (1.391–2.917)	< 0.001
ACEI vs. No RASI	1.284 (0.602–2.736)	0.516	1.307 (0.873–1.955)	0.192
ARB vs. No RASI	0.569 (0.281–1.155)	0.119	0.648 (0.459–0.915)	0.014
*Three year clinical outcomes*				
Primary endpoint (Death or MI)				
ACEI vs. ARB	1.276 (1.024–1.591)	0.030	1.174 (1.013–1.361)	0.032
ACEI vs. No RASI	1.470 (1.131–1.910)	0.004	1.225 (1.058–1.419)	0.006
ARB vs. No RASI	1.151 (0.883–1.501)	0.297	1.043 (0.904–1.202)	0.560
All-cause death				
ACEI vs. ARB	1.280 (0.973–1.684)	0.077	1.122 (0.935–1.347)	0.214
ACEI vs. No RASI	1.681 (1.230–2.297)	0.001	1.335 (1.119–1.593)	0.001
ARB vs. No RASI	1.312 (0.957–1.800)	0.091	1.189 (1.001–1.414)	0.049
Myocardial infarction: MI				
ACEI vs. ARB	1.332 (0.953–1.862)	0.093	1.333 (1.061–1.674)	0.013
ACEI vs. No RASI	1.141 (0.740–1.758)	0.549	1.099 (0.866–1.394)	0.437
ARB vs. No RASI	0.856 (0.555–1.322)	0.485	0.824 (0.658–1.031)	0.091
Stroke				
ACEI vs. ARB	1.756 (1.180–2.612)	0.005	1.521 (1.175–1.969)	0.001
ACEI vs. No RASI	1.165 (0.677–2.004)	0.581	1.056 (0.798–1.397)	0.701
ARB vs. No RASI	0.663 (0.392–1.121)	0.125	0.694 (0.536–0.897)	0.005

RASI, renin angiotensin system inhibitor; IPTW, inverse probability of treatment weighting; ACEI, angiotensin converting enzyme inhibitor; ARB, angiotensin receptor blocker

### Subgroup analysis

**Fig. 1:**
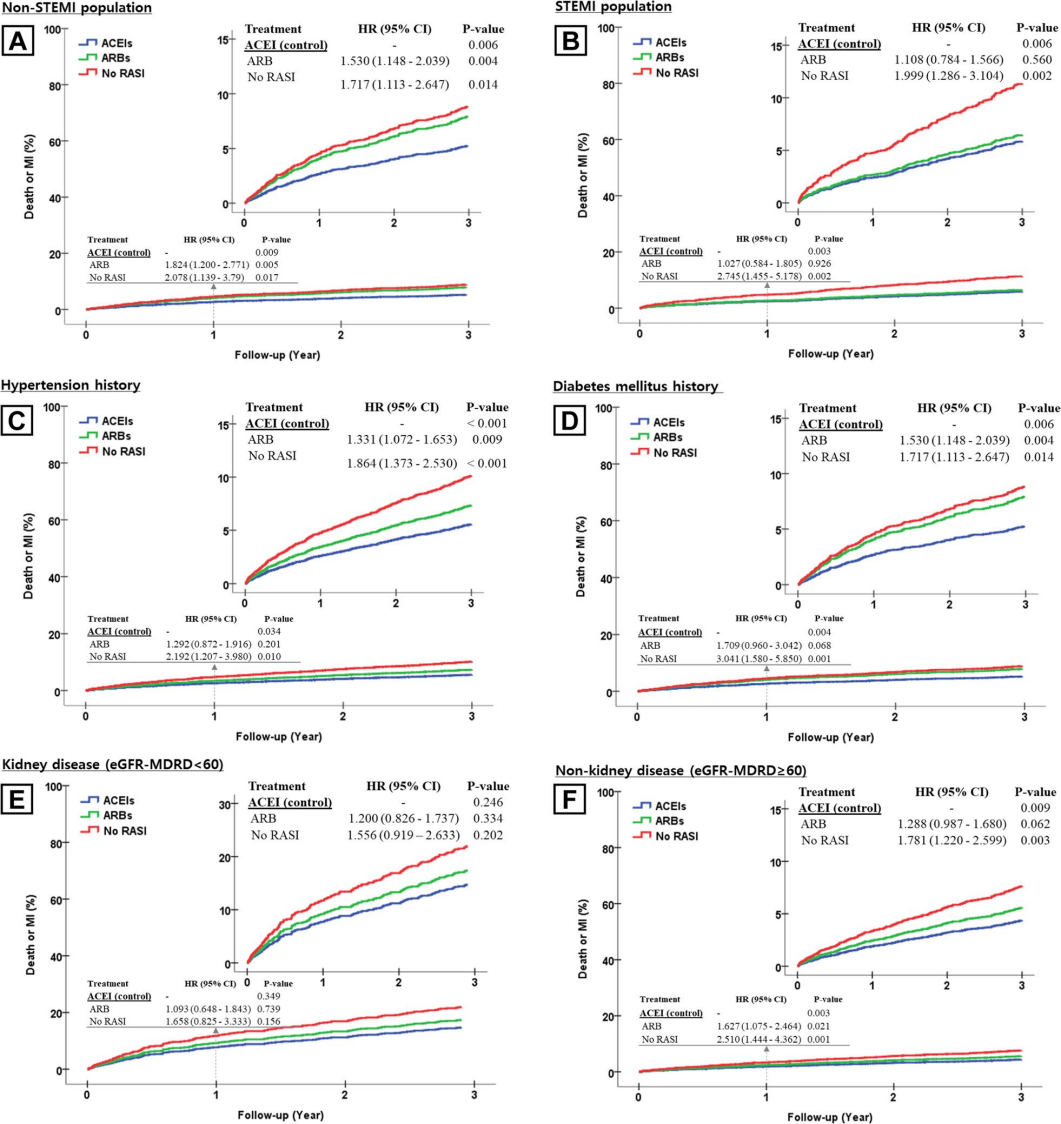
Inverse probability of treatment weighting score adjusted survival curves from Cox-proportional hazards models for death or myocardial infarction (MI) according to discharge treatment in overall and various subgroups. **a** Non-STEMI population, **b** STEMI population, **c** hypertension history population, **d** diabetes mellitus population, **e** kidney disease population, **f** non-kidney disease population. ACEI, angiotensin converting enzyme inhibitor; ARB, angiotensin receptor blocker; STEMI, ST segment elevation myocardial infarction; RASI, renin angiotensin system inhibitor; eGFR-MDRD, estimates glomerular filtration rate-the original modification of diet in renal disease

We calculated the adjusted HR for total death or MI in various complex subgroups at 3 years and 1 year after AMI (Fig. 1). Non- ST elevation MI (NSTEMI) patients who were treated with ACEIs during the 3 years after MI tended to have better clinical outcomes than NSTEMI patients treated with ARBs (ARBs compared with ACEIs, HR 1.530, 95% CI 1.148–2.039, *P* = 0.004, Fig. 1a). Additionally, for NSTEMI patients, ACEIs were more effective than ARBs when used during the first year of treatment, and equivalent thereafter (ARBs compared with ACEIs, HR 1.824, 95% CI 1.200–2.771, *P* = 0.005, Fig. 1a). There were no significant difference between the use of ARB at discharge and cardiac death or MI in other subgroups (STEMI, hypertension and diabetes mellitus, normal renal function) at 1 year (Fig. 1b–d, f). However, in the group of hypertension and diabetes mellitus, the clinical outcomes of the ARB group was tend to be worse than those of the ACEI group after adjustment at 3 years. In patients with kidney disease, there were no significant differences among the three groups with regard to cardiac death or MI at both 1 and 3 years (Fig. 1e).

### Switching medication

**Fig. 2:**
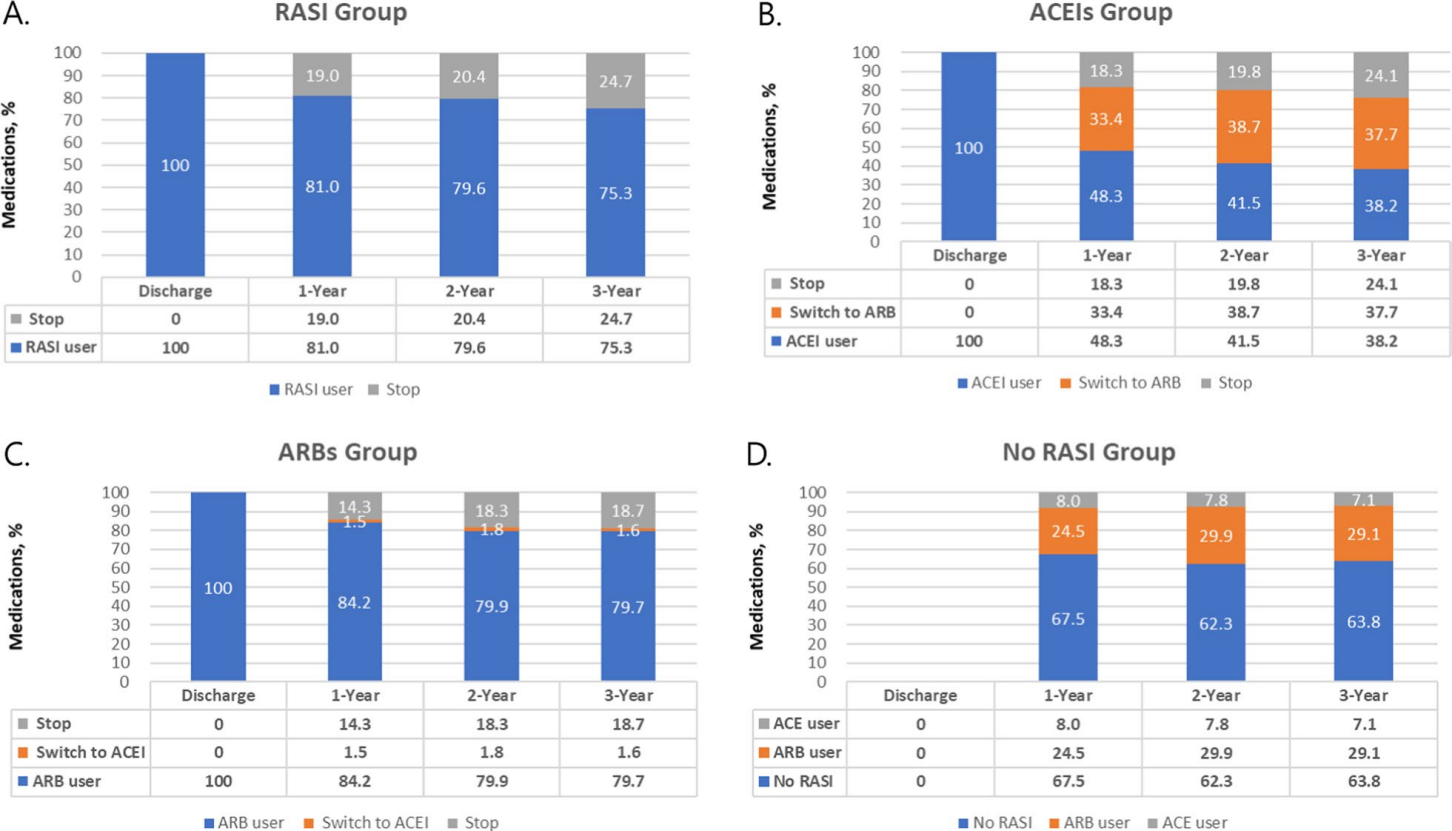
Changes in the use of renin-angiotensin system inhibitors after myocardial infarction in the Korean real-world. **a** All patients were required to remain on treatment, but 19% of patients receiving treatment with RASI stopped medication in the first year, **b** eighteen percent of patients receiving ACEIs stopped treatment in the first year and 33.4% of patients receiving ACEIs changed medication to ARBs, **c** fourteen percent of patients receiving ARBs stopped treatment in this real-world registry, while only about 1.5% switched from ARBs to ACEIs, **d** in patients not receiving RASI treatment, 24.5% began receiving ARBs and 8% began receiving ACEIs. RASI, renin-angiotensin system inhibitors; ACEI, angiotensin converting enzyme inhibitor; ARB, angiotensin receptor blocker; STEMI, ST segment elevation myocardial infarction

All patients were required to remain on treatment, but 19% of patients receiving treatment with RASI stopped medication in the first year (Fig. 2a). Interestingly, 18.3% of patients receiving ACEIs stopped treatment, and 14.3% of patients receiving ARBs stopped treatment in this real-world registry (Fig. 2b, c). Thirty three percent of patients receiving ACEIs switched to ARBs during the first year, while only about 1.5% switched from ARBs to ACEIs. In patients not receiving RASI treatment, 24.5% began receiving ARBs and 8% began receiving ACEIs (Fig. 2d). In total, 1,628 patients among 6536 patients either stopped treatment or were not receiving treatment after 1 year. From 1 year after AMI, 1151 patients received ACEIs for the duration of the study, 1,110 patients received ACEIs during the first year but switched to ARBs after, and 1,687 patients used ARBs for the duration of the study. Three hundred seventeen patients began not receiving any treatment but initiated treatment with ARBs after 1 year. Less than 2% of patients started, stopped, or changed treatment after the first year. Additional analysis for the primary outcomes was performed according to the timing of switch from each medications.

**Fig. 3:**
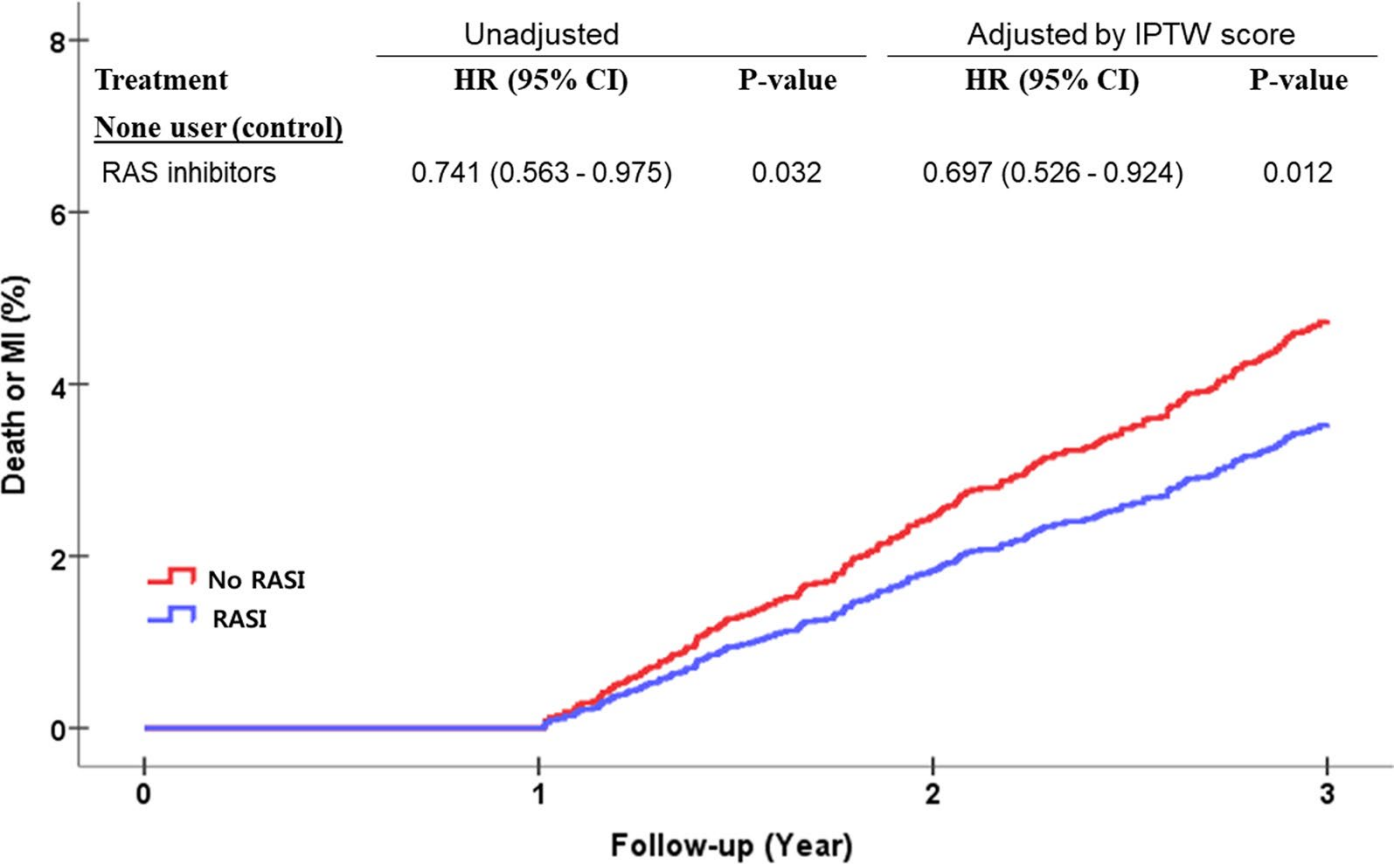
Survival curves from Cox-proportional hazards models for death or myocardial infarction (MI) according to renin-angiotensin system inhibitors treatment between 1-year to 3-year after an acute myocardial infarction. RASI, renin-angiotensin system inhibitors; ACEI, angiotensin converting enzyme inhibitor; ARB, angiotensin receptor blocker

**Fig. 4:**
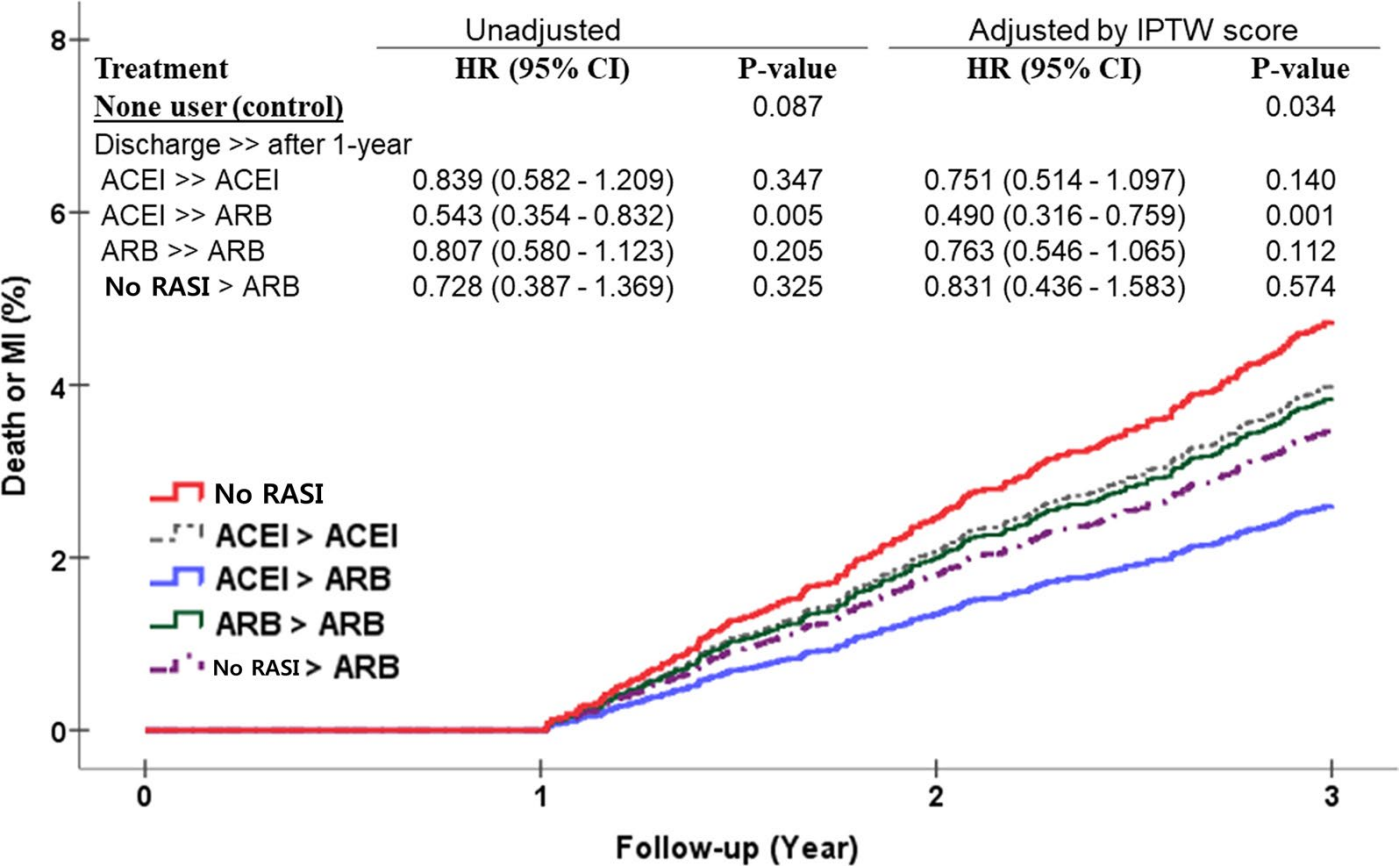
Survival curves from Cox-proportional hazards models for death or myocardial infarction (MI) according to switching renin-angiotensin system inhibitors treatment between 1-year to 3-year after an acute myocardial infarction. RASI, renin-angiotensin system inhibitors; ACEI, angiotensin converting enzyme inhibitor; ARB, angiotensin receptor blocker

When Landmark analysis was performed from 1 year to the end of the study period (Fig. 3), groups receiving RASI showed a 31% adjusted reduction in risk of death or recurrence of MI compared to patients not receiving RASI after adjustment with IPTW (HR, 0.741; 95% CI, 0.563–0.975; *P* = 0.012). While all groups who received RASI showed a statistically significant reduction in death or recurrence of AMI, patients who received ACEIs in the first year but switched to ARBs after 1 year were associated with statistically significant reduction in the incidence of death or recurrent AMI, while the other treatment groups did not differ from each other significantly (Fig. 4).

## Discussion

The main findings of our study are as follows. First, in AMI patients with preserved LV systolic function who underwent PCI, the risk of total death or recurrent AMI was lower in patients treated with ACEIs in the first year compared to patients treated with ARBs. Both groups showed lower risk compared to patients who did not receive any RASI. Second, for NSTEMI patients, treatment with ACEIs in the first year was associated with a greater reduction in risk compared to ARBs, but both were generally equivalent in risk reduction in patients with STEMI, hypertension, and diabetes mellitus. This suggests that NSTEMI patients should be treated with ACEIs in the first year. Third, AMI patients with preserved LV systolic function had a poorer prognosis if RASI were stopped after 1 year, suggesting that treatment should be continued beyond 1 year after AMI. Fourth, after 1 year, about a third of patients on ACEIs switched to ARBs, likely due to side effects, but this change in treatment did not alter their prognosis or risk of total death or recurrent AMI at least up to 3 years. This suggests that after the first year, switching the medication does not impact on long term major clinical outcomes. However, continuation of treatment is necessary.

Most randomized trials have demonstrated that ACEI therapy with captopril, enalapril, ramipril, trandolapril, or zofenopril started within 24 h to 16 days following an AMI improves the LV ejection fraction at one month to 1 year [3, 5, 17–19]. In these studies, the great majority of patients had an STEMI; data are limited in patients with a NSTEMI [10]. In addition, most patients were treated with either fibrinolytic therapy or no reperfusion; data in patients who underwent PCI, particularly treated with DESs for AMI are limited. ACEIs have also been reported to be beneficial in low risk patients after AMI [20], although the role of routine long term treatment in low risk patients who have undergone revascularization after STEMI is less certain. Therefore, the guidelines recommend that ACE inhibitors should be considered in all patients after STEMI in the absence of contraindications. [21]

Yang et al. showed that ARB had beneficial effects in terms of cardiac death or MI, which were comparable with those achieved with ACEIs in patients with STEMI with preserved LV systolic function [22]. They studied 6698 patients with STEMI who underwent PCI and had a LV ejection fraction ≥ 40% among the same Korean registry used in this study, but with data from a different time period (Between November 2005 and September 2010). Yang’s study differs from our study in that they enrolled only STEMI patients with EF greater than 40%, and they used a different time period and different duration of progress observation (Yang followed patients for 1 year, whereas we followed patients for 3 years). Yang’s study examined patients from a 5-year period (2005–2010) with EF greater than 40%, whereas our study examined patients from a different 5-year period (2011–2016) with EF greater than 50%, which reflect on the clinical outcomes of current contemporary PCI with DESs. In other words, our study examined a different set of lower-risk patients and examined longer-term outcomes by following them for 3 years instead of 1 year. It is notable that despite of these differences, both studies found that STEMI patients have no difference in the incidence of total death or recurrent AMI in groups treated with either ACEI or ARB at their 1-year progress observations. However, in the NSTEMI group (which was not analyzed by Yang et al.), our study found that NSTEMI patients who were treated with ACEIs during the first year after MI was associated with better clinical outcomes than NSTEMI patients treated with ARBs, suggesting the possibility that ACEIs may be more effective in the NSTEMI group in the first year than ARBs, although this requires further research. There are few studies examining RASI in NSTEMI patients. Kim et al. demonstrated that ACEIs showed more prominent ability to decrease the occurrences of MACEs, any repeat revascularization, and TVR compared to the ARB group in NSTEMI patients during a 2-year follow-up period. Additionally Byun et al. found that compared to ARB, ACEIs did not impact on causes of death, cardiac death, or recurrence of MI, but ACEIs did reduce the incidence of revascularization and MACE in diabetic NSTEMI patients [1, 23]. However, their study’s population included all NSTEMI patients, including patients with reduced EF. Our study only looked at AMI patients with EF greater than 50%, and suggests that the ACEI group had better outcomes than the ARB group in the first year. However, in our study, 33% of patients switched from ACEIs to ARBs during the first year, and patients may have changed drugs as early as the three to six-month points if they experienced side effects of ACEI, so it is not possible to conclude with certainty that one is more effective than the other.

Although the participants in our study had AMI with preserved LV systolic function, they also had a risk of LV remodeling post MI. LV remodeling is one of the major determinants of survival after recovery from MI, and the mechanism of LV remodeling within a few weeks after myocardial infarct reperfusion is well known[24]. The three main biomechanisms that contribute to the increase in the LV chamber volume post MI are (1) expansion of the infarct in the subacute phase, (2) subsequent non-ischemic infarct extension into the adjacent non-infarcted region, and (3) hypertrophy and dilatation of the non-infarcted myocardium in the chronic phase. Previous studies have led to a general consensus that treatment with ACEIs and ARBs has an anti-fibrotic effect on collagen formation in infarcted and remote non-infarcted regions [25]. This is the rationale for implementing drug therapy as soon as possible after MI, and the current ACC/AHA guidelines recommend ACE inhibitors as Class I drugs for use within the first 24 h after MI. When considering the obvious clinical benefits of ACEIs and ARBs, this suggests that there may be excessive of fibrosis of the infarct in untreated patients, when a smaller, more compact scar is preferred. Although our study cannot determine the superiority of either ACEIs or ARBs, the clinical results of ACEIs tend to be superior to those of ARBs. A plausible hypothesis for this is that these two drugs (ACEIs and ARBs), which are in the same system in terms of RAS inhibition, have different mechanisms of action[26]. ACEIs inhibit the conversion of angiotensin I to angiotensin II, which reduces the level of serum angiotensin II and activates the bradykinin system. In contrast, ARBs may cause prolonged elevation of angiotensin II levels and the upregulation of angiotensin I. It is well known that elevated serum levels of angiotensin II play an important role in the pathogenesis of coronary artery disease [27]. In addition, elevated angiotensin II levels may direct an increase in serum cholesterol levels via the interaction with macrophage AT1 receptors, stimulating 3-hydroxy-3-methylglutaryl coenzyme A reductase gene expression, ultimately leading to cholesterol accumulation in macrophages and foam cell formation [28].

Some data showed that treatment with ACEIs or ARBs after AMI was associated with improved long-term survival compared to a group not receiving treatment, regardless of underlying renal function, and was accompanied by low rates of adverse renal events [29]. The findings of our study support earlier studies that showed patients with kidney disease receiving RASI treatment after MI have better outcomes than patients not receiving RASI treatment. Although, the statistical significance was not confirmed, but risk of death or MI were better in both the ACEI and ARB groups than in the No RASI group. In all cases, our results suggest that RASI are effective and should be used in patients following AMI with preserved LV systolic function.

There is much controversy over the MI paradox when using ARB. Angiotensin II receptor stimulation may be less beneficial than previously proposed and may even be harmful under certain circumstances through mediation of growth promotion, fibrosis, and hypertrophy, as well as proatherogenic and proinflammatory effects. However, in a total of 19 of 24 studies of data on AMI, representing a total of 31 569 patients, use of ARBs was not associated with an increased risk of MI compared with placebo (odds ratio [OR] 0.94, 95% CI 0.75 to 1.16) nor when compared with ACE inhibitors (OR 1.01, 95% CI 0.87 to 1.16). Our data showed that 33% of patients using ACEIs changed to ARBs at the end of a year, and these patients showed an equal or improved prognosis compared to those who remained on ACEIs. This suggests that ARBs are safe and effective in a population with AMI and preserved LV function, particularly AMI patients undergoing PCI with DESs.

## Limitations

First, our study did not have data on LV function at follow-up, meaning that our study cannot account for whether patient’s outcomes were impacted by changes in LV function or if they regained or lost LV function during the study period. Because it is possible that RASI have different effectiveness in populations with greater or reduced EF, it will be necessary to examine this more in the future to confirm these findings. Second, because the registry data is non-randomized, it is possible there was selection bias. Although we performed an IPWT matched analysis to account for these potential confounding factors, it was not possible to correct for unmeasured variables. For example, our database does not include information on why physicians switched patients from ACEIs to ARBs. In the future, it will be necessary to conduct randomized trials to confirm and expand the findings of this study. Third, adverse clinical events were not centrally adjudicated in our registry, and were instead identified by the patient’s physician and confirmed by the principal investigator of each hospital. As a result, it is possible that some adverse events were not captured in the database.

## Data Availability

All data can be checked by sending an email to Correspondence.

## Abbreviations

AMI: Acute myocardial infarction
ACEI: Angiotensin-converting enzyme inhibitor
ARB: Angiotensin receptor blocker
LV: Left ventricular
RASI: Renin-angiotensin system inhibitor
PCI: Percutaneous coronary intervention
TLR: Target lesion revascularization
TVR: Target vessel revascularization
IPTW: Inverse probability of treatment weighting
